# Novel imaging techniques for hydroxychloroquine retinopathy

**DOI:** 10.3389/fmed.2022.1026934

**Published:** 2022-10-13

**Authors:** Imran H. Yusuf, Peter Charbel Issa, Seong Joon Ahn

**Affiliations:** ^1^Oxford Eye Hospital and Nuffield Department of Clinical Neurosciences, John Radcliffe Hospital, University of Oxford, Oxford, United Kingdom; ^2^Department of Ophthalmology, Hanyang University Hospital, Hanyang University College of Medicine, Seoul, South Korea

**Keywords:** hydroxychloroquine retinopathy, retinal imaging, optical coherence tomography, fundus autofluorescence, novel technique

## Abstract

Hydroxychloroquine retinopathy is an increasingly recognized cause of iatrogenic, irreversible visual impairment due to the expanding use of hydroxychloroquine in combination with improvements in disease detection following advances in retinal imaging techniques. The prevalence of disease is estimated to be greater than 5% amongst individuals who have used the drug for 5 years or more. In addition to conventional imaging modalities, such as spectral-domain optical coherence tomography (OCT) and fundus autofluorescence (FAF), novel retinal imaging techniques such as *en face* OCT, OCT angiography, fluorescence lifetime imaging ophthalmoscopy, quantitative autofluorescence, and retromode imaging are capable of detecting structural changes in the retina. These novel retinal imaging techniques have shown promise in detecting earlier disease than is possible with current mainstream imaging modalities. Moreover, these techniques may identify disease progression as well as enabling functional correlation. In the future, these novel imaging techniques may further reduce the risk of visual loss from hydroxychloroquine retinopathy through the earlier detection of pre-clinical disease.

## Introduction

Hydroxychloroquine retinopathy is a well-recognized toxic retinopathy resulting from exposure to hydroxychloroquine, a drug widely used for the treatment of rheumatologic and dermatologic disorders, such as systemic lupus erythematosus and rheumatoid arthritis (1–3). Hydroxychloroquine retinopathy may lead to irreversible, progressive visual loss in long-term users of hydroxychloroquine, particularly if the retinopathy is detected in the symptomatic phase when damage to the retinal pigment epithelium (RPE) is observed (4). Early detection of pre-symptomatic disease through retinal monitoring or screening is imperative to reduce the risk of visual loss in long-term hydroxychloroquine users who are increasing in number due to the proven systemic benefits of the drug, and expanding indications for its use (5).

Retinal imaging is particularly important for the early detection of hydroxychloroquine retinopathy. Indeed, the advent of spectral-domain optical coherence tomography (SD-OCT) has significantly improved the detection of retinopathy (6). In the optical coherence tomography (OCT) era, the prevalence of retinopathy has been estimated at over 5% using OCT-based diagnostics in long-term users (7, 8), having previously been estimated at less than 1% before the advent of SD-OCT (9). In recent years, retinal imaging techniques have been favored in the early detection of hydroxychloroquine retinopathy as they are objective, widely available, reproducible and time efficient when compared to functional tests (i.e., visual field testing, electroretinography).

Despite advances in retinal imaging, the detection of early hydroxychloroquine retinopathy remains challenging. However, novel retinal imaging techniques may enable the detection of disease at an earlier stage than is possible with current mainstream retinal imaging techniques. Accordingly, this review aims to summarize current data obtained from the application of novel imaging techniques and advanced image analysis in patients with hydroxychloroquine retinopathy, and considers the prospects for earlier disease detection in the future using these emerging techniques.

## Conventional imaging used for screening hydroxychloroquine retinopathy

Fundus photography and clinical examination techniques are routine, relatively low-cost tests performed in ophthalmology clinics. Currently, fundus examination and photography are not recommended as the only standard screening tests for hydroxychloroquine retinopathy (4). This is mainly due to the lack of sensitivity in the detection of early disease as fundus examination/photography can only detect severe retinopathy once RPE changes have developed. Diagnosing early retinopathy requires the identification of pre-fundoscopic signs (10). Nevertheless, the American Academy of Ophthalmology (AAO) guidelines (2016) recommend color fundus photography at baseline (within 1 year of hydroxychloroquine use) in order to exclude or document other pre-existing retinal or macular diseases (4).

Hydroxychloroquine retinopathy has been defined by the AAO as two or more tests demonstrating abnormalities consistent with toxicity, at least one of which should be objective. The Royal College of Ophthalmologists (RCOphth) guideline (2020) defines “definite retinopathy” as two abnormal tests consistent with disease, in order to reduce the risk of inappropriate cessation of the drug (4, 5). The RCOphth do not directly specify the objectivity of the tests, although since monitoring is undertaken with SD-OCT and fundus autofluorescence (FAF) imaging primarily, at least one objective test will be supportive in each case. The two recommendations differ in their screening protocols. The AAO suggests SD-OCT, FAF, multifocal electroretinography (mfERG), and/or automated visual field testing for annual screening, whereas the RCOphth recommends annual SD-OCT and FAF, with visual field testing reserved for those with a structural abnormality detected on retinal imaging. Most studies in literature use SD-OCT and FAF as part of monitoring protocols (4–6, 11–14), due to their ability to detect objective characteristic findings suggestive of hydroxychloroquine toxicity.

**FIGURE 1:**
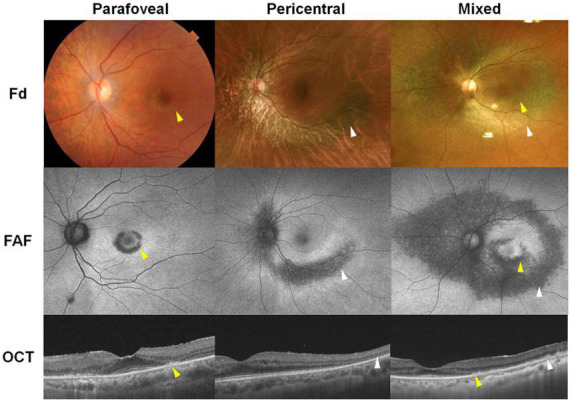
Representative images of hydroxychloroquine retinopathy in patients treated with hydroxychloroquine therapy (left: 200 mg/day for 21 years, middle: 200 mg/day for 20 years, right: 200 mg/day for 20 years). These cases demonstrate parafoveal, pericentral, and mixed (both) involvement on fundus examination (Fd), fundus autofluorescence (FAF), and optical coherence tomography (OCT) images. Yellow and white arrowheads indicate parafoveal and pericentral retinal damage, respectively.

The typical findings of hydroxychloroquine retinopathy observed on SD-OCT and FAF imaging as described in previous studies are presented in Table 1 (4–6, 11, 15–19). Typical abnormalities of hydroxychloroquine retinopathy on SD-OCT and FAF—the most commonly used mainstream clinical imaging modalities–are loss or attenuation of outer retinal layers (e.g., the ellipsoid zone) and thinning or attenuation of the RPE/Bruch’s membrane complex on OCT imaging (Figure 1), and/or pericentral or parafoveal hyperautofluorescence/hypoautofluorescence (Figure 1) on FAF imaging. SD-OCT has become the most important and frequently used screening modality as it is widely available in ophthalmology clinics and enables non-invasive, detailed assessment of structural changes in the retina. Accordingly, both the AAO and RCOphth recommended OCT as a primary test for screening (4, 5). Outer nuclear thinning without overt qualitative photoreceptor defects, which have been suggested as an early change in hydroxychloroquine retinopathy (17), is usually difficult to discern based on the examination of raw OCT B-scans, even by retinal specialists. However, methods of automated image analysis or longitudinal comparisons of sequential OCT images may assist in the identification of more subtle signs of toxic retinopathy (11, 20).

**TABLE 1:** Findings, advantages, and limitations of the conventional, widely available imaging modalities used for screening hydroxychloroquine retinopathy.

Modality	Findings	Advantages	Limitations
Spectral domain optical coherence tomography (OCT) (widely available)	• Reduced reflectivity of the ellipsoid zone • Disruption or loss of the photoreceptor layers and/or RPE thinning or loss in the parafoveal or pericentral areas • Outer nuclear layer thinning	• Accurate, objective assessment of the structural changes in the retina • Definitive, strong evidence if photoreceptor loss or outer retinal thinning is present in a typical (parafoveal or pericentral) pattern	• Early changes such as outer nuclear layer thinning without overt photoreceptor defects may be difficult to identify. • Focal damage may not be captured depending on the location of OCT scan. The whole extent of retinal damage cannot be captured within a single image. • Missed detection of pericentral changes when using conventional scan (6 mm in length or 20 degrees)
Fundus autofluorescence (FAF) (widely available)	• Hyper- or hypoautofluorescent patch or ring in the parafoveal or pericentral areas	• Provides a topographic view of damage across the fundus • Whole extent of retinal damages can be captured in a single wide-field image. • RPE involvement evident as hypoautofluorescence • Easier staging than with OCT	• Can be subjective when evaluating subtle or early abnormalities, leading to normal or ambiguous findings. Changes may not be detected as early as with SD-OCT in eyes with early changes • Greatly affected by media opacity, such as vitreous opacity and cataract, compared with OCT

Fundus autofluorescence is also a very useful imaging modality in hydroxychloroquine retinopathy as it is capable of capturing the topographic distribution of retinopathy with a single image (21). Furthermore, RPE damage, appearing as hypoautofluorescence on FAF imaging, can be easily determined on FAF images, enabling the classification of the severity of retinopathy, with severe stage defined as RPE loss on FAF imaging (Table 1; 6, 21, 22). However, FAF has limitations in the detection of early retinopathy since abnormalities may be subtle or absent in early disease (18, 22). In addition, FAF imaging, similar to other fundus imaging modalities, may be affected by cataract, vitreous and other media opacities, leading to poor image quality and hence difficulties in image interpretation (22).

## Advanced optical coherence tomography-based analysis

Several novel OCT-based techniques and methods of image analysis have been developed and used to evaluate retinal diseases. Some of these techniques have also been evaluated for the detection and monitoring of hydroxychloroquine retinopathy (Table 2).

**TABLE 2:** Novel imaging techniques used for evaluation of hydroxychloroquine retinopathy in the literature.

Category	Imaging modality/techniques (availability)	Findings	Advantages	References
Optical coherence tomography techniques	*En face* imaging (some availability)	• Changes in reflectivity in areas with photoreceptor defects	• Evaluation of whole extent of retinopathy using a single image • Quantitative measurement of area of photoreceptor defects • Quantitative evaluation of disease progression over time	(22)
	OCT angiography (some availability)	• Decreased mean vascular density in the deep capillary plexus layer • A large number of signal void zones in the choriocapillaris	• Potential additional parameter of retinal toxicity	(15, 27, 28)
	Topographic thickness maps (some availability)	• Characteristic (i.e., parafoveal and pericentral) patterns of retinal thinning	• Automated visualization of abnormal retinal thickness (retinal thinning) • Monitoring of disease progression using the areas of retinal thinning	(11, 16)
	Wide-field OCT (limited availability)	• Outer retinal defects in the peripheral retina	• Potential for earlier detection of pericentral retinopathy	(32, 33)
	Sequential retinal thickness (widely available)	• Decrease in retinal thickness preceding the appearance of qualitative morphologic changes	• Preclinical detection of drug toxicity	(20, 24)
	Minimum intensity analysis (limited availability)	• Increased reflectivity in eyes with hydroxychloroquine retinopathy	• Reported to have high sensitivity and specificity	(34)
Fundus autofluorescence techniques	Fluorescence lifetime imaging ophthalmoscopy (FLIO) (limited availability)	• Significantly enhanced FLIO lifetime in the damaged regions	• Detection of retinal toxicity at very early stages	(36–38)
	Near-infrared fundus autofluorescence (some availability)	• Reduced parafoveal or pericentral near-infrared fundus autofluorescence	• Better visualization of deeper structures such as retinal pigment epithelium or better images in eyes with media opacities	(6)
	Quantitative autofluorescence (limited availability)	• Increased autofluorescence intensity	• More objective interpretation of FAF findings	(43, 44, 46)
Others under investigation	Adaptive optics (limited availability)	• Disruption of the cone photoreceptor mosaic, • Decreased cone density in the inferior parafoveal area • An irregular cone density on the areas without obvious photoreceptor defects	• Early detection of photoreceptor damage	(47, 48)
	Microperimetry (limited availability)	• Contiguous scotoma points in the parafoveal region	• Superior specificity when compared with mfERG • Potential to match functional defects topographically with structural defects	(51–53)
	Retromode imaging (limited availability)	• Parafoveal or pericentral ring-shaped or round areas of decreased reflectance with prominent deep choroidal vessels	• Potential additional parameter of retinal toxicity	(56)

### *En face* technologies

**FIGURE 2:**
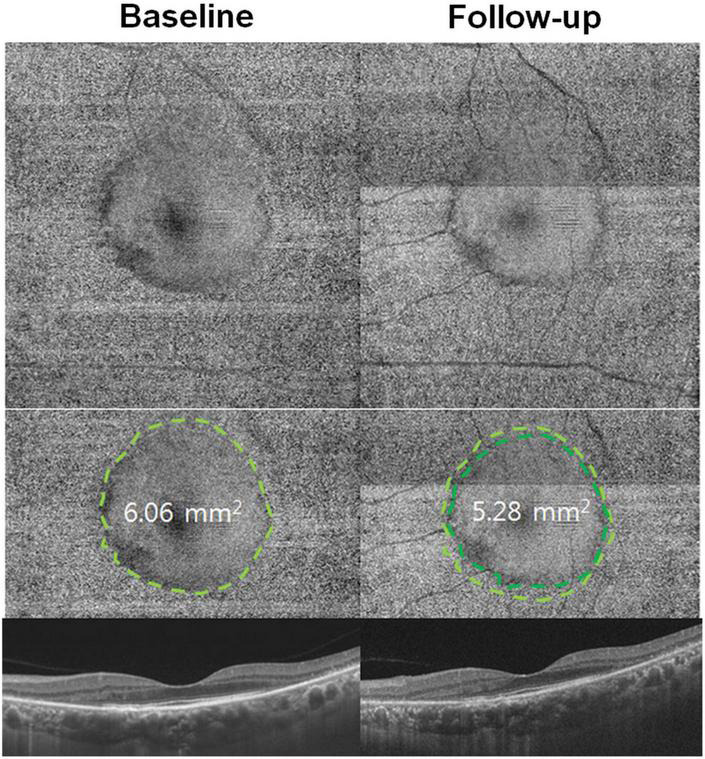
*En face* optical coherence tomography (OCT) imaging in hydroxychloroquine retinopathy. *En face* OCT images demonstrate a smooth surface in the central area (inside the dashed line) on the areas with intact photoreceptors, whereas, the more granular reflectivity (outside the line) is noted in the pericentral area with retinal degenerative changes. The area with intact photoreceptors can be calculated and used as a quantitative measure of photoreceptor damage. Progression of photoreceptor damage is represented as a constricted ring at follow-up (10 months later). The quantitative measure, area with intact photoreceptors, decreases from 6.06 at baseline to 5.28 mm^2^ at the follow-up visit [Reprinted with permission from Ahn et al. (23)].

Using *en face* technology, OCT images perpendicular to the scanning axis, called C-scans, can be obtained (reconstructed) from three-dimensional volumetric data. As the depth or layer of the retina for *en face* imaging can be chosen by the investigator, the resulting *en face* images are similar to fundus photographs but with the benefit of exhibiting the information about the depth or layer of interest. *En face* OCT imaging encompasses wider retinal areas than conventional OCT B-scans, leading to immediate interpretation of the topography of changes occurring over a relatively wide field (17, 23). A recent study on *en face* ellipsoid zone (EZ) imaging for hydroxychloroquine retinopathy showed uneven reflectivity in areas with photoreceptor defects, whereas areas with intact photoreceptors appeared smoother (Figure 2; 23). The extent of retinopathy can be evaluated using a single *en face* OCT image, which may represent a more sensitive method of defining the distribution of early disease when compared to FAF imaging. However, further studies are required to directly compare their utility in early hydroxychloroquine retinopathy. Furthermore, the affected area can be quantified and used to evaluate disease progression over time (Figure 2), using sequential measurements derived from *en face* OCT imaging.

For *en face* OCT, segmentation can be modified using different slabs according to the retinal layer of interest. In hydroxychloroquine retinopathy, EZ line topography may be particularly useful in the detection of retinal toxicity since changes in the EZ are characteristic on cross-sectional OCT B-scan images (23, 24). Furthermore, since outer retinal thinning is a characteristic early feature of hydroxychloroquine retinopathy, other outer retinal layers can be isolated for en face image analysis in order to potentially detect more subtle, early markers of toxicity.

### Optical coherence tomography angiography

Optical coherence tomography angiography (OCTA) employs motion contrast imaging to obtain high-resolution volumetric blood flow information for the generation of angiographic images (25). It is a quick and non-invasive technique that has the clinical capability of localizing and delineating pathology, along with the ability to show both structural and blood flow information (25, 26). Although OCTA has a more obvious potential application in the evaluation of retinal vascular disease, this imaging technique has been evaluated in patients taking hydroxychloroquine. High-risk patients (with >5 years of hydroxychloroquine exposure) showed lower retinal vascular density and flow rates, and a wider foveal avascular zone than low-risk patients (hydroxychloroquine duration < 5 years) (27). Accordingly, Bulut et al. (27) suggested that OCTA evaluation has the potential to detect hydroxychloroquine-induced retinal toxicity at an early stage. A recent study showed that the mean vascular density in the deep capillary plexus layer decreased in those showing abnormal mfERG recordings, indicating a potential role of OCTA in the early detection of retinopathy (28). Moreover, another recent report showed a large number of signal void zones in the choriocapillaris of patients with hydroxychloroquine retinopathy, which were more remarkable in those with severe disease (15). The authors suggested an association between choriocapillaris involvement and disease progression after drug cessation, although the underlying mechanism is unclear (15).

However, it is questionable whether OCTA has any advantage over conventional OCT imaging for retinopathy screening. The role of OCTA in the early detection of retinopathy, together with the effect of hydroxychloroquine on the retinal and choroidal vasculature, should be explored in further studies.

### Topographic thickness maps

In addition to the characteristic outer retinal defects on OCT B-scan images, outer retinal thinning is another hallmark feature of hydroxychloroquine retinopathy. Although easily overlooked during a clinician’s qualitative evaluation of B-scan images, total, inner, and outer retinal thickness maps can be generated using automated segmentation, and the areas with retinal thickness changes compared to age-matched controls can be visualized (16). Using this technique, outer retinal thickness maps demonstrated a characteristic parafoveal pattern of outer retinal thinning in eyes with parafoveal hydroxychloroquine retinopathy, whereas, the inner retina showed no focal defects (16). Based on the spatial characteristics of hydroxychloroquine-induced retinal thinning, topographic maps are likely to represent clinically valuable tools in the identification of early toxicity. In addition to identifying the area of outer retinal thinning, the map could be used to monitor disease progression.

**FIGURE 3:**
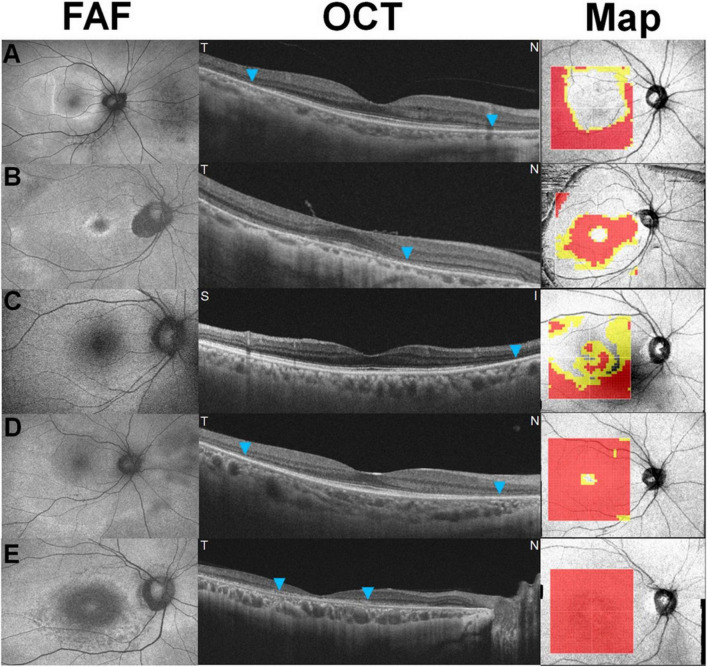
Five recognizable patterns [a pericentral ring **(A)**, parafoveal ring **(B)**, mixed ring **(C)**, central island **(D)**, and whole macular thinning **(E)**] of retinal thinning in retinal thickness deviation maps on swept-source optical coherence tomography in eyes with hydroxychloroquine retinopathy. Yellow (representing a thickness of <5% of the normative level) or red pixels (representing a thickness of <1%) indicate retinal thinning [Reprinted with permission from Kim et al. (11)].

**FIGURE 4:**
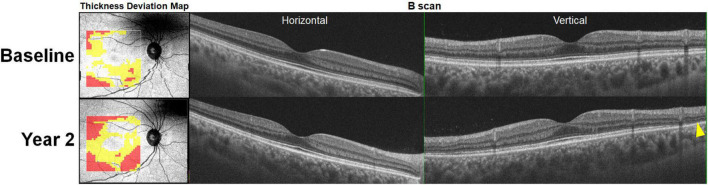
Retinal thinning noted on optical coherence tomography (OCT) retinal thickness deviation maps (left) at baseline (hydroxychloroquine [200 mg/day] use for 8 years) prior to the development of recognizable photoreceptor defects (yellow arrowhead) on OCT B-scan images noted 2 years later. At the follow-up visit, the thickness deviation map indicates more extensive retinal thinning in the inferior area.

A recent study showed extensive retinal thinning in five recognizable patterns on a deviation map (Figure 3) based on whole retinal thickness in eyes with hydroxychloroquine retinopathy (11). The map showed excellent sensitivity in the detection of retinopathy (over 95% in two independent sets of patients) and early detection of retinopathy before the development of recognizable photoreceptor changes on OCT B scans (Figure 4), which further highlighted the clinical usefulness of topographic maps for hydroxychloroquine retinopathy screening. With advances in OCT image quality and more reliable segmentation algorithms, topographic maps obtained by segmentation of specific retinal layers may further improve suitability for screening or evaluation of hydroxychloroquine retinopathy.

### Sequential retinal thickness

Some recent data suggest that careful and sequential measurement of regional retinal thickness changes may, in some instances, provide clues to very early stages of damage (24). Sequential thickness changes in parafoveal and pericentral retinopathy showed marked retinal thinning in parafoveal and perifoveal areas, respectively. The rapid decrease in retinal thickness preceded the appearance of any obvious qualitative morphologic changes on OCT B-scans; therefore, clinicians might identify drug-induced retinal toxicity early using these techniques (24). A recent study showed that 38 of 82 patients with rapid retinal thinning eventually developed conventional OCT or visual field signs of retinal toxicity (20). Accordingly, if validated, this characteristic pattern in the early natural history of retinal toxicity may be used to detect retinopathy at an earlier stage (e.g., 4–5 years before clinical diagnosis) than is possible with standard OCT image analysis (24). Further, rates of change of retinal thickness may be utilized for evaluation of retinopathy progression (29).

### Wide-field scans

Wide-field imaging is now extensively used for various retinal diseases, such as diabetes mellitus, particularly to examine and document changes occurring in the peripheral retina (30). A recent guideline from the International Widefield Imaging Study group recommended that the term “widefield” be limited to areas of the retina beyond the posterior pole but posterior to the vortex vein ampulla in all four quadrants, and that the term “ultra-widefield” should describe retinal anatomic features anterior to the vortex vein ampullae in all four quadrants (31).

Several instruments provide widefield spectral domain or swept-source OCT imaging. The currently available OCT technology can capture up to 23 mm in a single scan, which may visualize retinal changes both in the posterior pole and beyond. This is particularly important for Asian patients, as retinal damage commonly occurs beyond the macular area in hydroxychloroquine retinopathy. Ahn et al. (32) suggested that 12 radial scans of 12-mm length could detect retinal damage in all pericentral cases, whereas conventional SD-OCT line scans of 6-mm length failed to demonstrate any outer retinal defects in approximately one-third of the Asian patients. Another report showed that wide field OCT imaging can improve the detection of peripheral retinal abnormalities associated with hydroxychloroquine toxicity (33). Wide-field OCT scans are therefore important in monitoring protocols in Asian patients (4, 29). Test protocols in other populations should ideally include a method of peripheral retinal examination (such as widefield FAF), since pericentral disease was identified in approximately 2% of Caucasian patients in one series (19).

The natural history of retinopathy in terms of whether the paracentral or pericentral area(s) or the more peripheral retina is involved first is unclear, since the peripheral retina has rarely been evaluated by OCT in this group. Since ultra-widefield FAF imaging showed significant peripheral degeneration in eyes with pericentral hydroxychloroquine retinopathy (22), the area of initial retinal damage might be further clarified using widefield or ultra-widefield OCT (33). Further investigation of the use of widefield OCT for hydroxychloroquine retinopathy is required to validate the usefulness of wide-field scans for hydroxychloroquine retinopathy screening and monitoring. If peripheral retinal degeneration occurs first, widefield OCT imaging may detect earlier signs of retinopathy undetectable by standard OCT scans.

### Minimum intensity analysis

The minimum intensity analysis of OCT is another novel post-acquisition analysis of each A-scan to identify the lowest image intensity value in the area between the inner limiting membrane and RPE (34). The lowest reflectivity (i.e. the minimum intensity) is usually measured in the outer nuclear layer. In hydroxychloroquine retinopathy, increased reflectivity or loss of the outer nuclear layer results in displacement of minimum intensity measures to the inner nuclear layer which has a higher reflectivity. Minimum intensity analysis may result in high sensitivity and specificity for detecting hydroxychloroquine retinopathy, although these diagnostic estimates were obtained with a small sample size and added value for very early macular changes remains to be addressed (34). However, this analysis is currently limited to specific OCT manufacturers, and its potential for use as a screening tool has not been fully validated.

## Fundus autofluorescence techniques

Lipofuscin is a fluorophore present in RPE cells that absorbs short-wavelength light with a peak excitation wavelength of 470 nm (blue) and emits fluorescence at a peak wavelength of approximately 630 nm (orange) (35). FAF is a non-invasive imaging modality widely used in evaluation of retinal diseases in real-world clinical practice, as it provides a density map of lipofuscin distribution at the ocular fundus (21).

**FIGURE 5:**
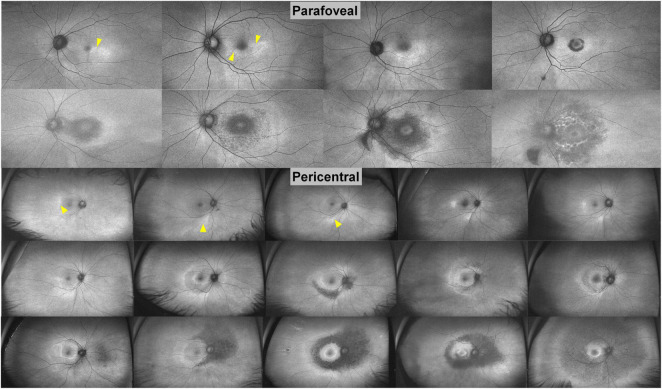
Ultra-widefield fundus autofluorescence images in patients with parafoveal and pericentral hydroxychloroquine retinopathy, placed in order of severity based on the extent of hyper- and hypo-autofluorescence. From a temporal or inferior patchy hyperautofluorescence (arrowheads) to extensive hypoautofluorescence, the extent of retinal damage increases significantly [Modified from Ahn et al. (22)].

Fundus autofluorescence is now widely used in the screening of hydroxychloroquine retinopathy as a standard test for identifying objective, structural damage caused by the drug (4, 5, 14, 18). The extent and severity of retinal damage in hydroxychloroquine retinopathy can be easily appreciated using ultrawide-field FAF, which can identify variable peripheral involvement in patients with pericentral retinopathy (Figure 5; 22). Accordingly, wide-field adaptations of FAF are recommended for Asian patients (22, 32). However, since pericentral disease may occur more rarely in other ethnic groups, some recommendations advise widefield FAF for all patients, where available (5). The extent of retinal toxicity on FAF images correlates with visual field results; therefore, functional predictions may be made based on the topographic distribution of disease on FAF imaging (22). Widefield FAF imaging is recommended by the RCOphth, where available, to capture pericentral disease. However, its utility for routine and standard screening is uncertain and requires further validation.

### Fluorescence lifetime imaging ophthalmoscopy

Fundus autofluorescence can characterize not only the spatial distribution of fluorescence intensity, but also the lifetime of fluorophores (such as lipofuscin) and the average time a fluorophore remains excited (36). Fluorescence lifetime imaging ophthalmoscopy (FLIO) is an emerging imaging modality for the *in vivo* measurement of the lifetime of endogenous retinal fluorophores (37). Patients with hydroxychloroquine toxicity showed a significantly enhanced FLIO lifetime in the damaged regions, typically in a pattern corresponding to degenerative retinal changes (38, 39). Detection of early toxicity appears to be feasible with FLIO, although the clinical benefits of FLIO have not been fully evaluated and the imaging technique is not yet widely available.

### Near-infrared fundus autofluorescence

**FIGURE 6:**
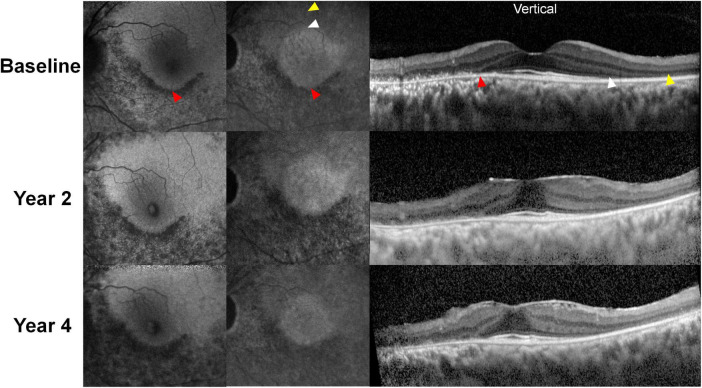
Representative blue and near-infrared autofluorescence images (BAF and NIA, respectively) and optical coherence tomography B-scan images over the 4-year follow-up period in a patient treated with hydroxychloroquine 300 mg per day for 13 years. Hypoautofluorescence was almost identical on BAF and NIA images, which correspond to the defective (thinned) retinal pigment epithelium/Bruch’s membrane complex line. However, NIA demonstrates a central hyperautofluorescent area, aligns to the area with intact photoreceptor layers (borders between the areas with and without photoreceptor defects demarcated by white arrowheads). In contrast, BAF showed no clear distinction between the areas with and without photoreceptor defects in the superior (S) parafoveal area. As the areas with intact photoreceptors decreased over time due to progression of retinopathy, the central hyperautofluorescence on NIA imaging decreased in size, whereas the superior parafoveal area in BAF shows no definite changes.

Near-infrared fundus autofluorescence (NIA) targets melanin as the endogenous fluorophore within the RPE and has also been evaluated in patients with chloroquine retinopathy (6). Mild cases with reduced parafoveal responses on mfERG showed reduced parafoveal NIA, which suggests its potential for the detection of early hydroxychloroquine retinopathy (6). Blue and near-infrared FAF images in an eye with severe hydroxychloroquine retinopathy are shown in Figure 6, demonstrating that the distinction between normal and defective photoreceptors may be more easily discernible with longer wavelength (787 nm) rather than short-wavelength (488 nm) FAF. Deeper structures such as RPE and choroid may be better visualized using NIA (40) and it may confer advantages as the primary tool for tracking disease progression over short-wavelength AF, given the increased patient comfort and cooperation during imaging (41).

### Quantitative autofluorescence

By using an internal reference for AF intensity normalization, quantitative fundus autofluorescence enables quantitative comparison of AF intensities (42–44). This technique has been utilized for the differentiation of bull’s eye maculopathy and other retinal disorders (42, 43, 45). A few recent reports showed utility of quantitative autofluorescence in the detection of retinal changes in hydroxychloroquine retinopathy (43–46). However, the usefulness of quantitative autofluorescence for early detection is questionable as possible effects are small. However in later disease stages, when effects are larger, OCT shows obvious changes.

## Others under investigations

### Adaptive optics

Adaptive optics (AO) allows direct visualization of individual photoreceptor cells (mainly cones). Accordingly, several authors have applied this imaging technique in the evaluation of hydroxychloroquine retinopathy (47, 48). Stepien et al. (48) demonstrated the disruption of the cone photoreceptor mosaic, which corresponded to the areas showing EZ defects on SD-OCT B-scan images. Interestingly, areas without obvious photoreceptor defects on SD-OCT also showed an irregular cone density in AO. Another study using AO revealed a decreased cone density in the inferior parafoveal area, which is a common site of initial damage in eyes without overt photoreceptor damage on OCT imaging, but not in the superior parafoveal area (47). These findings suggest the potential of AO for the early detection of photoreceptor damage caused by hydroxychloroquine toxicity. However, AO equipment is not readily accessible and the imaging is difficult to perform, analyze, and interpret. Thus, this is currently not relevant to real-world screening. Moreover, it is unlikely that changes in the cone mosaic within the macula (where high-quality AO imaging is usually performed) would be discernible in patients with pericentral disease. Further studies are required to validate its use in hydroxychloroquine retinopathy.

### Microperimetry

Microperimetry assesses the pointwise retinal sensitivity in the macula by integrating computerized threshold perimetry with real-time fundus imaging (49). Since this modality allows precise localization of functional defects, it has the potential to match function and structure in eyes with hydroxychloroquine retinopathy (50). A few reports have evaluated microperimetry in the detection of hydroxychloroquine retinopathy (51, 52). Iftikhar et al. (53) showed inferior sensitivity but superior specificity of microperimetry when compared with mfERG, using the criterion of three or more contiguous scotoma points in the parafoveal region to define disease. From the superior specificity, microperimetry was suggested as an ancillary test to exclude the diagnosis if the screening tests provided conflicting results (e.g., structural tests showing no structural defects but mfERG revealing positive findings), but microperimetry showed negative results (51, 52). However, microperimetry is not widely available, although it may be a useful tool in patients with possible early structural defects and uncertain automated visual field test results.

### Retro-mode imaging

Retro-mode imaging is a non-invasive retinal imaging technique that enables visualization of the outer retina using scanning laser ophthalmoscopy with pseudo-3D images, including shadows. This imaging technique has advantages in the visualization of pathologic changes in the outer retina and has been evaluated in age-related macular degeneration, myopic foveoschisis, and central serous chorioretinopathy (54, 55). A recent study showed that patients with hydroxychloroquine retinopathy demonstrated parafoveal or pericentral ring-shaped or round areas of decreased reflectance with prominent deep choroidal vessels, with 100% sensitivity (56). However, the possibility of false positives should be carefully considered, particularly in eyes with high myopia. The authors suggested that imaging is useful for the early detection of hydroxychloroquine retinopathy; however, the limited availability of imaging would prevent the widespread use of this technique for hydroxychloroquine retinopathy screening (56).

## Future directions and conclusion

In the future, a greater understanding of the early natural history of hydroxychloroquine retinopathy as revealed by retinal imaging techniques is likely to enable the earlier detection of disease. For example, the identification of sequential retinal thinning within the macula in some patients who were subsequently diagnosed with retinopathy by conventional definitions (20) illustrates how basic image analysis may further inform risk of toxicity, beyond the inspection of a single OCT B-scan by a trained human observer. The widespread availability of analysis of segmented images may further refine the predictive value of sequential or cross-sectional images by identifying changes in specific retinal layers, rather than total retinal thickness.

Further work is required to determine the relative sensitivities and specificities of all diagnostic modalities at different stages of retinopathy. These data, in particular, will define the early natural history of hydroxychloroquine retinopathy. For example, sequential retinal thickness measurements appear to be in part predictive of the development of retinopathy by current definitions (20). Other novel imaging modalities may further qualify the early natural history of retinopathy (i.e., that precede visual field changes). This may enable the prediction of the development of definite retinopathy by current definitions, enabling the redirection of resources toward those at highest risk of developing retinopathy.

A more objective definition of early retinopathy through data from these imaging modalities may facilitate the management of confirmed cases of retinopathy. Currently, recommendations suggest that written communication is provided to the prescribing physician and patient about the certainty and severity of retinopathy in order to enable a decision to be made about treatment cessation or continuation. Although most patients elect to stop drug therapy, some patients with severe systemic symptoms/disease may elect to continue hydroxychloroquine, particularly if the retinopathy is mild, given that the disease progresses slowly. Current dosing guidelines suggest a dose less than 5 mg per kilogram of absolute body weight reduces the risk of developing retinopathy, although there is no absolutely safe dose by body weight (7). It is unclear whether a reduced daily dose of hydroxychloroquine at the point of disease detection slows down the progression of retinopathy.

Artificial intelligence (AI) might provide further predictive value through the analysis of retinal imaging studies of individuals exposed to hydroxychloroquine prior to the development of clinically detectable retinopathy, as it has been shown to predict the risk of future development of type 2 diabetes mellitus, and/or chronic renal failure (57). AI analysis of OCT images may be able to identify earlier signs of toxic retinopathy than are identifiable using basic methods of image analysis available in supporting software, for example, by detecting more focal changes or patterns, or indeed novel features that have not been yet associated with toxic retinopathy. By identifying the earliest changes, AI may help to further clarify the natural history of very early structural retinopathy prior to the “early” retinopathy as defined by the current classification. The threshold might be defined as the degree of retinal structural change that would prevent any meaningful changes in retinal function. In this way, AI might more accurately elucidate the early natural history of retinopathy enabling the detection of cases that might reach this threshold and to support a more precise definition of hydroxychloroquine retinopathy. Accordingly, AI may help to determine the interval for repeated screening with respect to a pre-defined level of risk set according to healthcare budgets for each given individual at risk, thereby reducing the cost of retinopathy screening. However, significant challenges exist in identifying a significantly large dataset on which to train and validate an algorithm.

In conclusion, hydroxychloroquine retinopathy is known to be more prevalent than previously estimated as advances in retinal imaging have enabled the early detection of retinopathy. As a growing number of patients taking the drug are at risk of vision loss, novel imaging techniques should be developed or applied for hydroxychloroquine retinopathy. These advances should be integrated into screening pathways to personalize care according to the individual risk of retinopathy based on a pre-defined level of accepted risk. The application of AI for screening hydroxychloroquine retinopathy and molecular imaging are likely to help reduce the risk of vision loss due to retinal toxicity by facilitating earlier detection of the disease.

## Author contributions

IY and SA: conception, design, and data collection. All authors: analysis, interpretation, obtain funding, overall responsibility, and approve the submitted version.

## References

[1] Alarcon GS McGwin G Bertoli AM Fessler BJ Calvo-Alen J Bastian HM Effect of hydroxychloroquine on the survival of patients with systemic lupus erythematosus: data from LUMINA, a multiethnic US cohort (LUMINA L). *Ann Rheum Dis.* (2007) 66:1168–72. 10.1136/ard.2006.068676 17389655 PMC1955128

[2] Fanouriakis A Kostopoulou M Alunno A Aringer M Bajema I Boletis JN 2019 update of the EULAR recommendations for the management of systemic lupus erythematosus. *Ann Rheum Dis.* (2019) 78:736–45. 10.1136/annrheumdis-2019-215089 30926722

[3] Rempenault C Combe B Barnetche T Gaujoux-Viala C Lukas C Morel J Clinical and structural efficacy of hydroxychloroquine in rheumatoid arthritis: a systematic review. *Arthritis Care Res.* (2020) 72:36–40. 10.1002/acr.23826 30629341

[4] Marmor MF Kellner U Lai TY Melles RB Mieler WF. Recommendations on screening for chloroquine and hydroxychloroquine retinopathy (2016 revision). *Ophthalmology.* (2016) 123:1386–94. 10.1016/j.ophtha.2016.01.058 26992838

[5] Yusuf IH Foot B Lotery AJ. The Royal College of Ophthalmologists recommendations on monitoring for hydroxychloroquine and chloroquine users in the United Kingdom (2020 revision): executive summary. *Eye.* (2021) 35:1532–7. 10.1038/s41433-020-01380-2 33423043 PMC8169737

[6] Kellner S Weinitz S Kellner U. Spectral domain optical coherence tomography detects early stages of chloroquine retinopathy similar to multifocal electroretinography, fundus autofluorescence and near-infrared autofluorescence. *Br J Ophthalmol.* (2009) 93:1444–7. 10.1136/bjo.2008.157198 19692385

[7] Melles RB Marmor MF. The risk of toxic retinopathy in patients on long-term hydroxychloroquine therapy. *JAMA Ophthalmol.* (2014) 132:1453–60. 10.1001/jamaophthalmol.2014.3459 25275721

[8] Jaumouille S Espargilliere D Mouriaux F Mortemousque B. [Clinical evaluation of the new screening procedures for hydroxychloroquine retinopathy, according to the American Academy of Ophthalmology Guidelines. Prospective study of 184 patients]. *J Francais Ophtalmologie.* (2015) 38:377–87. 10.1016/j.jfo.2015.01.005 25913441

[9] Wolfe F Marmor MF. Rates and predictors of hydroxychloroquine retinal toxicity in patients with rheumatoid arthritis and systemic lupus erythematosus. *Arthritis Care Res.* (2010) 62:775–84. 10.1002/acr.20133 20535788

[10] Marmor MF. The demise of the bull’s eye (screening for hydroxychloroquine retinopathy). *Retina.* (2016) 36:1803–5. 10.1097/IAE.0000000000001151 27388736

[11] Kim KE Ahn SJ Woo SJ Park KH Lee BR Lee YK Use of optical coherence tomography retinal thickness deviation map for hydroxychloroquine retinopathy screening. *Ophthalmology.* (2021) 128:110–9. 10.1016/j.ophtha.2020.06.021 32553941

[12] Yates M Malaiya R Stack J Galloway JB. Hydroxychloroquine use: the potential impact of new ocular screening guidelines. *Eye.* (2018) 32:161–2. 10.1038/eye.2017.166 28799559 PMC5770712

[13] Gobbett A Kotagiri A Bracewell C Smith J. Two years’ experience of screening for hydroxychloroquine retinopathy. *Eye.* (2021) 35:1171–7. 10.1038/s41433-020-1028-4 32636495 PMC8115138

[14] Marshall E Robertson M Kam S Penwarden A Riga P Davies N. Prevalence of hydroxychloroquine retinopathy using 2018 Royal College of Ophthalmologists diagnostic criteria. *Eye.* (2021) 35:343–8. 10.1038/s41433-020-1038-2 32587388 PMC7316164

[15] Ahn SJ Ryu SJ Lim HW Lee BR. Toxic effects of hydroxychloroquine on the choroid: evidence from multimodal imaging. *Retina.* (2019) 39:1016–26. 10.1097/IAE.0000000000002047 29373341

[16] de Sisternes L Hu J Rubin DL Marmor MF. Localization of damage in progressive hydroxychloroquine retinopathy on and off the drug: inner versus outer retina, parafovea versus peripheral fovea. *Invest Ophthalmol Vis Sci.* (2015) 56:3415–26. 10.1167/iovs.14-16345 26024126 PMC4455312

[17] Lally DR Heier JS Baumal C Witkin AJ Maler S Shah CP Expanded spectral domain-OCT findings in the early detection of hydroxychloroquine retinopathy and changes following drug cessation. *Int J Retina Vitreous.* (2016) 2:18. 10.1186/s40942-016-0042-y 27847636 PMC5088472

[18] Marmor MF. Fundus autofluorescence is not the best early screen for hydroxychloroquine toxicity. *JAMA Ophthalmol.* (2013) 131:1487–8. 10.1001/jamaophthalmol.2013.4835 24232086

[19] Melles RB Marmor MF. Pericentral retinopathy and racial differences in hydroxychloroquine toxicity. *Ophthalmology.* (2015) 122:110–6. 10.1016/j.ophtha.2014.07.018 25182842

[20] Melles RB Marmor MF. Rapid macular thinning is an early indicator of hydroxychloroquine retinal toxicity. *Ophthalmology.* (2022) 129:1004–13. 10.1016/j.ophtha.2022.05.002 35568277

[21] Yung M Klufas MA Sarraf D. Clinical applications of fundus autofluorescence in retinal disease. *Int J Retina Vitreous.* (2016) 2:12. 10.1186/s40942-016-0035-x 27847630 PMC5088473

[22] Ahn SJ Joung J Lee BR. Evaluation of hydroxychloroquine retinopathy using ultra-widefield fundus autofluorescence: peripheral findings in the retinopathy. *Am J Ophthalmol.* (2020) 209:35–44. 10.1016/j.ajo.2019.09.008 31526798

[23] Ahn SJ Joung J Lee BR. En face optical coherence tomography imaging of the photoreceptor layers in hydroxychloroquine retinopathy. *Am J Ophthalmol.* (2019) 199:71–81. 10.1016/j.ajo.2018.11.003 30448463

[24] Marmor MF Durbin M de Sisternes L Pham BH. Sequential retinal thickness analysis shows hydroxychloroquine damage before other screening techniques. *Retin Cases Brief Rep.* (2021) 15:185–96. 10.1097/ICB.0000000000001108 33394957

[25] Kashani AH Chen CL Gahm JK Zheng F Richter GM Rosenfeld PJ Optical coherence tomography angiography: a comprehensive review of current methods and clinical applications. *Prog Retin Eye Res.* (2017) 60:66–100. 10.1016/j.preteyeres.2017.07.002 28760677 PMC5600872

[26] Spaide RF Fujimoto JG Waheed NK. Image artifacts in optical coherence tomography angiography. *Retina.* (2015) 35:2163–80. 10.1097/IAE.0000000000000765 26428607 PMC4712934

[27] Bulut M Akidan M Gozkaya O Erol MK Cengiz A Cay HF. Optical coherence tomography angiography for screening of hydroxychloroquine-induced retinal alterations. *Graefes Arch Clin Exp Ophthalmol.* (2018) 256:2075–81. 10.1007/s00417-018-4117-3 30159602

[28] Akhlaghi M Kianersi F Radmehr H Dehghani A Naderi Beni A Noorshargh P. Evaluation of optical coherence tomography angiography parameters in patients treated with hydroxychloroquine. *BMC Ophthalmol.* (2021) 21:209. 10.1186/s12886-021-01977-5 33975575 PMC8112017

[29] Ahn SJ Seo EJ Kim KE Kim YJ Lee BR Kim JG Long-term progression of pericentral hydroxychloroquine retinopathy. *Ophthalmology.* (2021) 128:889–98. 10.1016/j.ophtha.2020.10.029 33129843

[30] Ghasemi Falavarjani K Tsui I Sadda SR. Ultra-wide-field imaging in diabetic retinopathy. *Vision Res.* (2017) 139:187–90. 10.1016/j.visres.2017.02.009 28688908

[31] Choudhry N Duker JS Freund KB Kiss S Querques G Rosen R Classification and guidelines for widefield imaging: recommendations from the International Widefield Imaging Study Group. *Ophthalmol Retina.* (2019) 3:843–9. 10.1016/j.oret.2019.05.007 31302104

[32] Ahn SJ Joung J Lim HW Lee BR. Optical coherence tomography protocols for screening of hydroxychloroquine retinopathy in Asian patients. *Am J Ophthalmol.* (2017) 184:11–8. 10.1016/j.ajo.2017.09.025 28964805

[33] Corradetti G Violanti S Au A Sarraf D. Wide field retinal imaging and the detection of drug associated retinal toxicity. *Int J Retina Vitreous.* (2019) 5(Suppl. 1):26. 10.1186/s40942-019-0172-0 31890286 PMC6907121

[34] Allahdina AM Stetson PF Vitale S Wong WT Chew EY Ferris FL III Optical coherence tomography minimum intensity as an objective measure for the detection of hydroxychloroquine toxicity. *Invest Ophthalmol Vis Sci.* (2018) 59:1953–63. 10.1167/iovs.17-22668 29677357 PMC5894928

[35] Delori FC Dorey CK Staurenghi G Arend O Goger DG Weiter JJ. In vivo fluorescence of the ocular fundus exhibits retinal pigment epithelium lipofuscin characteristics. *Invest Ophthalmol Vis Sci.* (1995) 36:718–29. 7890502

[36] Sauer L Vitale AS Modersitzki NK Bernstein PS. Fluorescence lifetime imaging ophthalmoscopy: autofluorescence imaging and beyond. *Eye.* (2021) 35:93–109. 10.1038/s41433-020-01287-y 33268846 PMC7852552

[37] Sauer L Andersen KM Li B Gensure RH Hammer M Bernstein PS. Fluorescence lifetime imaging ophthalmoscopy (FLIO) of macular pigment. *Invest Ophthalmol Vis Sci.* (2018) 59:3094–103. 10.1167/iovs.18-23886 30025128 PMC6009392

[38] Solberg Y Dysli C Moller B Wolf S Zinkernagel MS. Fluorescence lifetimes in patients with hydroxychloroquine retinopathy. *Invest Ophthalmol Vis Sci.* (2019) 60:2165–72. 10.1167/iovs.18-26079 31108547

[39] Sauer L Calvo CM Vitale AS Henrie N Milliken CM Bernstein PS. Imaging of hydroxychloroquine toxicity with fluorescence lifetime imaging ophthalmoscopy. *Ophthalmol Retina.* (2019) 3:814–25. 10.1016/j.oret.2019.04.025 31345727

[40] Keilhauer CN Delori FC. Near-infrared autofluorescence imaging of the fundus: visualization of ocular melanin. *Invest Ophthalmol Vis Sci.* (2006) 47:3556–64. 10.1167/iovs.06-0122 16877429

[41] Jauregui R Park KS Duong JK Sparrow JR Tsang SH. Quantitative comparison of near-infrared versus short-wave autofluorescence imaging in monitoring progression of retinitis pigmentosa. *Am J Ophthalmol.* (2018) 194:120–5. 10.1016/j.ajo.2018.07.012 30053465 PMC6165697

[42] Duncker T Tsang SH Lee W Zernant J Allikmets R Delori FC Quantitative fundus autofluorescence distinguishes ABCA4-associated and non-ABCA4-associated bull’s-eye maculopathy. *Ophthalmology.* (2015) 122:345–55. 10.1016/j.ophtha.2014.08.017 25283059 PMC4306619

[43] Reichel C Berlin A Radun V Tarau IS Hillenkamp J Kleefeldt N Quantitative fundus autofluorescence in systemic chloroquine/hydroxychloroquine therapy. *Transl Vis Sci Technol.* (2020) 9:42. 10.1167/tvst.9.9.42 32934892 PMC7463177

[44] Greenstein VC Lima de Carvalho JR Jr Parmann R Amaro-Quireza L Lee W Hood DC Quantitative fundus autofluorescence in HCQ retinopathy. *Invest Ophthalmol Vis Sci.* (2020) 61:41. 10.1167/iovs.61.11.41 32976563 PMC7521180

[45] Gliem M Muller PL Birtel J Herrmann P McGuinness MB Holz FG Quantitative fundus autofluorescence and genetic associations in macular, cone, and cone-rod dystrophies. *Ophthalmol Retina.* (2020) 4:737–49. 10.1016/j.oret.2020.02.009 32646556

[46] Parrulli S Cozzi M Airaldi M Romano F Viola F Sarzi-Puttini P Quantitative autofluorescence findings in patients undergoing hydroxychloroquine treatment. *Clin Exp Ophthalmol.* (2022) 50:500–9. 10.1111/ceo.14090 35503294 PMC9545387

[47] Babeau F Busetto T Hamel C Villain M Daien V. Adaptive optics: a tool for screening hydroxychloroquine-induced maculopathy? *Acta Ophthalmol.* (2017) 95:e424–5. 10.1111/aos.13276 27805308

[48] Stepien KE Han DP Schell J Godara P Rha J Carroll J. Spectral-domain optical coherence tomography and adaptive optics may detect hydroxychloroquine retinal toxicity before symptomatic vision loss. *Trans Am Ophthalmol Soc.* (2009) 107:28–33. 20126479 PMC2814561

[49] Pfau M Jolly JK Wu Z Denniss J Lad EM Guymer RH Fundus-controlled perimetry (microperimetry): application as outcome measure in clinical trials. *Prog Retin Eye Res.* (2021) 82:100907. 10.1016/j.preteyeres.2020.100907 33022378 PMC12872260

[50] Rohrschneider K Bultmann S Springer C. Use of fundus perimetry (microperimetry) to quantify macular sensitivity. *Prog Retin Eye Res.* (2008) 27:536–48. 10.1016/j.preteyeres.2008.07.003 18723109

[51] Jivrajka RV Genead MA McAnany JJ Chow CC Mieler WF. Microperimetric sensitivity in patients on hydroxychloroquine (Plaquenil) therapy. *Eye.* (2013) 27:1044–52. 10.1038/eye.2013.112 23764990 PMC3772354

[52] Martinez-Costa L Victoria Ibanez M Murcia-Bello C Epifanio I Verdejo-Gimeno C Beltran-Catalan E Use of microperimetry to evaluate hydroxychloroquine and chloroquine retinal toxicity. *Can J Ophthalmol.* (2013) 48:400–5. 10.1016/j.jcjo.2013.03.018 24093187

[53] Iftikhar M Kaur R Nefalar A Usmani B Kherani S Rashid I Microperimetry as a screening test for hydroxychloroquine retinopathy: the hard-risk-1 study. *Retina.* (2019) 39:485–91. 10.1097/IAE.0000000000002313 30234854 PMC6391179

[54] Tanaka Y Shimada N Ohno-Matsui K Hayashi W Hayashi K Moriyama M Retromode retinal imaging of macular retinoschisis in highly myopic eyes. *Am J Ophthalmol.* (2010) 149:635–40.e1. 10.1016/j.ajo.2009.10.024 20346779

[55] Shin YU Lee BR. Retro-mode Imaging for retinal pigment epithelium alterations in central serous chorioretinopathy. *Am J Ophthalmol.* (2012) 154:155–63.e4. 10.1016/j.ajo.2012.01.023 22503695

[56] Ahn SJ Lee SU Lee SH Lee BR. Evaluation of retromode imaging for use in hydroxychloroquine retinopathy. *Am J Ophthalmol.* (2018) 196:44–52. 10.1016/j.ajo.2018.08.013 30118686

[57] Zhang K Liu X Xu J Yuan J Cai W Chen T Deep-learning models for the detection and incidence prediction of chronic kidney disease and type 2 diabetes from retinal fundus images. *Nat Biomed Eng.* (2021) 5:533–45. 10.1038/s41551-021-00745-6 34131321

